# Complete defect in PA-PLA_1_α secretion function leading to autosomal recessive woolly hair and hypotrichosis: insights from a novel compound heterozygous *LIPH* variant study in a Chinese pedigree

**DOI:** 10.3389/fgene.2025.1591409

**Published:** 2025-05-09

**Authors:** Xinyue Zhang, Kexin Guo, Jiawei Liu, Xueting Yang, Rui Zhang, Rongrong Wang, Donglai Ma, Xue Zhang

**Affiliations:** ^1^ McKusick-Zhang Center for Genetic Medicine, State Key Laboratory for Complex Severe and Rare Diseases, Institute of Basic Medical Sciences Chinese Academy of Medical Sciences, School of Basic Medicine Peking Union Medical College, Beijing, China; ^2^ Department of Prenatal Diagnosis, Women’s Hospital of Nanjing Medical University, Nanjing Women and Children’s Healthcare Hospital, Nanjing, China; ^3^ State Key Laboratory for Complex Severe and Rare Diseases, Chinese Academy of Medical Science and Peking Union Medical College, National Clinical Research Center for Dermatologic and Immunologic Diseases, Beijing, China

**Keywords:** woolly hair, hypotrichosis, ARWH, LIPH, missense variant

## Abstract

Autosomal recessive woolly hair/hypotrichosis (ARWH) is a rare inherited hair disease. In this study, we report a 31-year-old Chinese female with the characteristic clinical features of woolly hair and hypotrichosis. Through whole-exome sequencing (WES), we identified a novel missense variant (NM_139248.3: c.530T>G: p.Leu177Arg) and a previously reported missense variant (c.742C>A: p.His248Asn) of *LIPH* in the patient. TA cloning demonstrated that these variants were located on different alleles, supporting an autosomal recessive inheritance pattern. *In silico* tools predicted the novel variant to be disease-causing, likely reducing the stability of PA-PLA_1_α, the protein encoded by *LIPH*. PA-PLA_1_α, a member of the AB hydrolase superfamily and the lipase family, functions as a secreted protein to perform its hydrolytic and catalytic activities. Through a secretion assay, we observed that the novel missense variant c.530T>G almost abolished the secretion of the variant protein compared to the control (*p* < 0.0001). The direct blocking of secretion has only been reported in two variants in previous studies. This means that it is likely to result in the complete loss of its hydrolytic function, which will eventually lead to the disease. Notably, all the variants that directly stopped secretion happened when the normal amino acid was replaced by arginine. This suggests that the arginine substitutions may be closely linked to making secretion less effective. Our study not only elucidates the genetic underlying in a Chinese patient with woolly hair but also clarifies its pathogenic mechanism. These discoveries may facilitate the advancement of future diagnostic and treatment approaches.

## 1 Introduction

Autosomal recessive woolly hair/hypotrichosis (ARWH) is a nonsyndromic hereditary hair disease first defined by Hutchinson in 1974 (Hutchinson et al., 1974). Disease onset typically occurs soon after birth and is clinically characterized by coarse, sparse, dry, and tightly curled hair, which may also involve beard and axillary hair (Kazantseva et al., 2006). Compared with other family members, patients usually have blonde or lighter hair (Horev et al., 2009). ARWH exhibits significant genetic heterogeneity, with causative variants identified in several genes. Based on these genes, ARWH is classified into three types: ARWH1 (OMIM #278150) caused by variants in *LPAR6*, ARWH2 (OMIM #604379) caused by variants in *LIPH*, and ARWH3 (OMIM #616760) caused by variants in *KRT25*. Additionally, homozygous nonsense variants in *C3orf52*, which interacts with LIPH, have also been detected in individuals with ARWH (Kazantseva et al., 2006; Pasternack et al., 2008; Shimomura et al., 2008; Ansar et al., 2015; Malki et al., 2020). Understanding the specific roles of these genes and the consequences of their variants is essential for elucidating the pathology of ARWH.

Among the causative genes of ARWH, the *LIPH* gene has received particular attention. Situated on chromosome 3q27-q28, *LIPH* spans approximately 45 kb and contains 10 exons (Jin et al., 2002). It encodes the enzyme lipase member H (phosphatidic acid-preferring phospholipase A1α, PA-PLA_1_α). This secreted enzyme works extracellularly, hydrolyzing phosphatidic acid (PA) to produce lysophosphatidic acid (LPA), which is an essential molecule for the growth of hair follicles (Horev et al., 2009). Consequently, the functional disruption of PA-PLA_1_α has been recognized as a key mechanism in ARWH2 pathogenesis. This disruption leads to deficient LPA production, which severely impairs normal hair development and results in the characteristic ARWH phenotype. Existing studies on pathogenic *LIPH* variants have predominantly focused on those impairing the hydrolytic activity of PA-PLA_1_α, whereas cases involving secretion defects are comparatively rare and remain less explored as a pathogenic mechanism. This leaves a gap in our understanding of ARWH pathogenesis, particularly secretion defects, which requires further study.

Here, we report a Chinese patient with clinical symptoms of woolly hair and hypotrichosis since birth. In this patient, we identified a novel variant (NM_139248.3: c.530T>G, p.Leu177Arg) and a reported variant (c.742C>A, p.His248Asn) in *LIPH*. Through genetic and functional analyses, we confirmed that this novel variant disrupts the development of hair follicles by completely abolishing PA-PLA_1_α secretion function, ultimately resulting in ARWH. Notably, this study found that arginine substitutions may lead to defective PA-PLA_1_α secretion, providing evidence for a previously unrecognized pathogenic mechanism. Finally, we systematically reviewed reported pathogenic variants of ARWH and their corresponding phenotypes across populations, revealing that *LIPH* variants are the predominant cause of ARWH in Chinese patients. This highlights the importance of prioritizing *LIPH* gene variants in the clinical diagnosis of Chinese patients with ARWH-like symptoms.

## 2 Methods

### 2.1 Clinical data collection and ethical compliance

This study enrolled patients who presented with distinct phenotypes of short, thin, and curly hair. The patient provided written informed consent prior to participation for the collection of her case details, images, and peripheral blood samples. The study received approval (Approval No. 2022170) from Peking Union Medical College’s Institutional Review Board.

### 2.2 Whole-exome sequencing (WES) and Sanger sequencing

Genomic DNA was extracted from the patient’s peripheral blood. The sequencing library was prepared. Target regions were sequenced with paired-end reads of 150 bp and 100× raw read coverage. The human reference genome (GRCh37/hg19) was used to align the produced raw reads. The variants were annotated with ANNOVAR and screened through public databases, including the dbSNP database, the 1000 Genomes Project, and gnomAD. Candidate pathogenic variants identified via WES were subsequently verified using Sanger sequencing. Primers were designed by Primer-BLAST online software. The results of the sequencing were examined by SnapGene software and then further validated.

### 2.3 Bioinformatics analysis

REVEL, CADD, and Mutation Assessor were used to predict pathogenicity. Evolutionary conservation analysis was performed using T-Coffee for multiple sequence alignments. Functionally important domains and sites within proteins were identified by referring to the InterPro database. The change in the stability of the variant protein sequence or structure was predicted using MUpro and iStable. The effect of the variant on protein structure was assessed using HOPE. AlphaFold was employed to construct a three-dimensional (3D) protein model, and PyMOL was used to visualize the protein structures of both the wild-type (WT) and variant.

### 2.4 RNA extraction, cDNA synthesis, and TA cloning construction

Using the conventional RNA extraction method, the RNA was isolated from the patient’s peripheral blood. Subsequently, the RNA was reverse-transcribed into cDNA according to the protocol. The sequence of the *LIPH* variants (c.530T>G and c.742C>A) was amplified from the above cDNA using the primer pair *LIPH*-RTPCR-F: 5′- CAG​ATG​TTG​GCA​GAA​GGA​GC -3′ and *LIPH*-RTPCR-R: 5′- GCTGACACACTGCCATC -3′. Following the normal procedure, RT-PCR products were cloned into the pMD18-T vector. Sequencing was performed on 50 clones chosen at random.

### 2.5 Cell culture, plasmid construction, and transfection

HEK293T cells were cultured in complete Dulbecco’s Modified Eagle Medium (DMEM) at 37°C under a 5% CO_2_ atmosphere. WT, as well as the c.530T>G and c.742C>A variants of *LIPH*, were linked into pEGFP-N vectors. HEK293T cells were transfected with Lipofectamine 3000 (Invitrogen, United States).

### 2.6 Quantitative reverse transcription-PCR (qPCR)

RNAs from HEK293T cells transfected with pEGFP-N vectors were reverse transcribed into cDNAs. qPCRs were performed using SYBR Green^®^ Master Mix (Yeasen, China). The primers for *LIPH* and the internal control *ACTB* used for qPCR were *LIPH*-qPCR-F: 5′- GTG​CTT​GTC​AAG​ATC​AGA​CGC-3′ and *LIPH*-qPCR-R: 5′- CGC​AGG​TCA​GGT​TTT​TCC​TTG-3′; *ACTB*-qPCR-F: 5′-CAT​GTA​CGT​TGC​TAT​CCA​GGC-3′; *ACTB*-qPCR-R: 5′-CTC​CTT​AAT​GTC​ACG​CAC​GAT-3′. Data were shown as the mean ± SD.

### 2.7 Preparation of supernatants and cell lysates

After transfection with pEGFP-N vectors for 24 h, the complete medium for HEK293T cells was replaced with serum-free medium. Following a 24-h incubation, the media were harvested and precipitated using methanol and chloroform. The precipitated protein pellet was redissolved in 40 μL of 1× SDS sample buffer with protease inhibitors (Roche, Switzerland) and phosphatase inhibitors (Roche, Switzerland) and boiled for 10 min. The remaining HEK293T cells were washed with DPBS. Then, 150 μL of 1× SDS sample buffer, as mentioned above, was added directly to the cell pellet and boiled for 10 min.

### 2.8 Western blotting

Western blotting was performed using a conventional method. Protein extracts from the supernatants and cell lysates were separated by SDS-PAGE. The primary antibodies used for probing the proteins were anti-β-Actin antibody (Cell Signaling Technology, 3700, United States) and anti-LIPH antibody (Proteintech, 16602-1-AP, China). The results were detected using chemiluminescence with species-specific HRP-conjugated secondary antibodies.

## 3 Results

### 3.1 Clinical features

**FIGURE 1:**
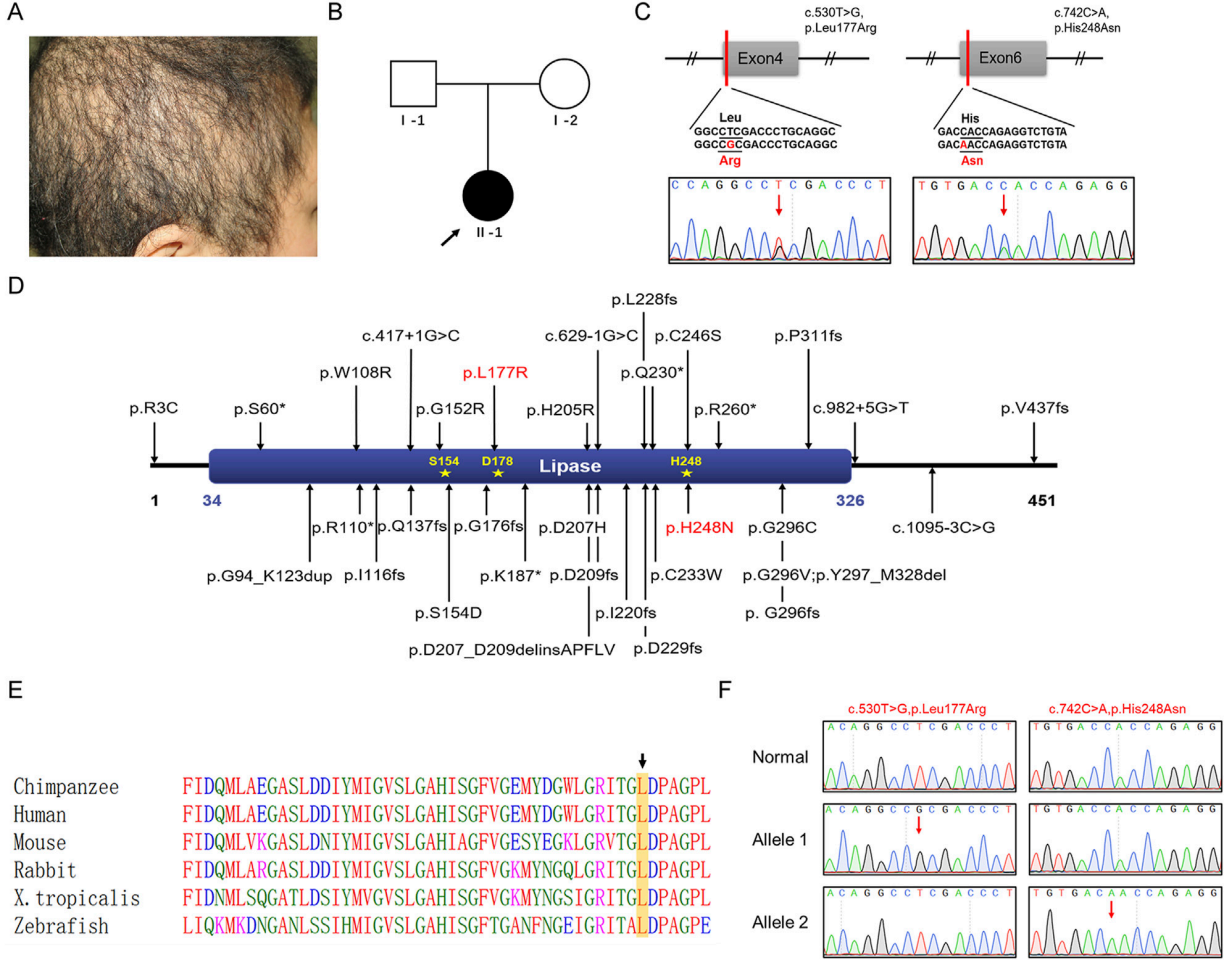
Clinical characteristics of a Chinese patient with ARWH and identification and analysis of the *LIPH* variants. **(A)** Clinical symptoms of the patient with short, thin, curly, and coarse hair. **(B)** Family pedigree: The black circle indicates the affected individual, and the arrow indicates the proband. **(C)** Sanger sequencing confirmed the c.530T>G and c.742C>A variants in *LIPH* in the patient. The two variant bases are marked by red arrows. **(D)** The positions of all reported pathogenic variants associated with ARWH are shown. The LIPH protein contained one crucial domain, the lipase (34–326) domain, and three active sites, Ser154, Asp178, and His248. Red denotes variants found in this work. **(E)** Leu177 was evolutionarily conserved across different species. Leucine is marked with a black arrow and framed in a yellow box. **(F)** TA cloning and sequencing indicated that the variants occurred in trans in the patient. The two variant bases are marked by red arrows.

The patient was a 31-year-old Chinese female who presented with short, thin, curly, and coarse hair that had been prone to breakage since birth (Figure 1A). She exhibited no abnormalities in intelligence or overall health and had no significant medical or family history of skin or limb deformities. Her parents were not consanguineous (Figure 1B) and declined further genetic testing.

### 3.2 Genomic sequencing and *in silico* analysis

WES was performed to identify pathogenic variants associated with the patient’s symptoms. Ultimately, two missense variants were identified in *LIPH* (NM_139248.3): c.530T>G (p.Leu177Arg) and c.742C>A (p.His248Asn). These variants were verified by Sanger sequencing (Figure 1C). The p.His248Asn variant was a known pathogenic variant associated with woolly hair/hypotrichosis. The p.Leu177Arg variant, on the other hand, was novel and had not been found in the dbSNP database, HGMD, or in-house databases (all reported pathogenic variants were shown in Figure 1D). Referring to the InterPro database, this novel variant residue is situated in a crucial domain known as lipase (IPR013818), very close to the active site (Asp178) (Figure 1D). This evidence suggests it has the potential to affect protein function by modifying the area surrounding the site. The leucine residue at amino acid position 177 is highly conserved across species (Figure 1E). The variant was predicted to be pathogenic by *in silico* analysis tools (REVEL, 0.983 damaging; Mutation Taster, 1 disease-causing; CADD, 29.7 pathogenic). The classification of the variant was “likely pathogenic” (PM1+PM2_supporting + PP3_strong) according to the ACMG/AMP 2015 guideline. In addition, no variants associated with woolly hair or hypotrichosis were found.

### 3.3 Located variants by TA cloning

The unavailability of peripheral blood samples from the patient’s parents precluded us from determining the origin of these variants. To verify whether each of the two variants was inherited separately from the father and the mother, TA cloning was employed. PCR was used to amplify an *LIPH* gene fragment encompassing the c.530T>G and c.742C>A variant sites from the patient’s genomic DNA. The PCR products were then cloned into the pMD18-T vector, and a total of 50 clones were randomly selected for subsequent Sanger sequencing. As shown in Figure 1F, the two variants occurred in trans (i.e., on different alleles), confirming that the patient had woolly hair and hypotrichosis with an autosomal recessive inheritance pattern.

### 3.4 Structural bioinformatics analysis of the variant impact

The possible impact of the p.Leu177Arg variant on the secondary and tertiary structures of PA-PLA_1_α was further investigated. The p.Leu177Arg variant was predicted to decrease the stability of the PA-PLA_1_α based on results from MUpro and iStable software. According to the HOPE database, the p.Leu177Arg variant led to an increase in amino acid size, a shift in charge from neutral to positive, and a loss of hydrophobic interactions within the core. These alterations may affect intramolecular interactions and subsequently influence the function of PA-PLA_1_α.

**FIGURE 2:**
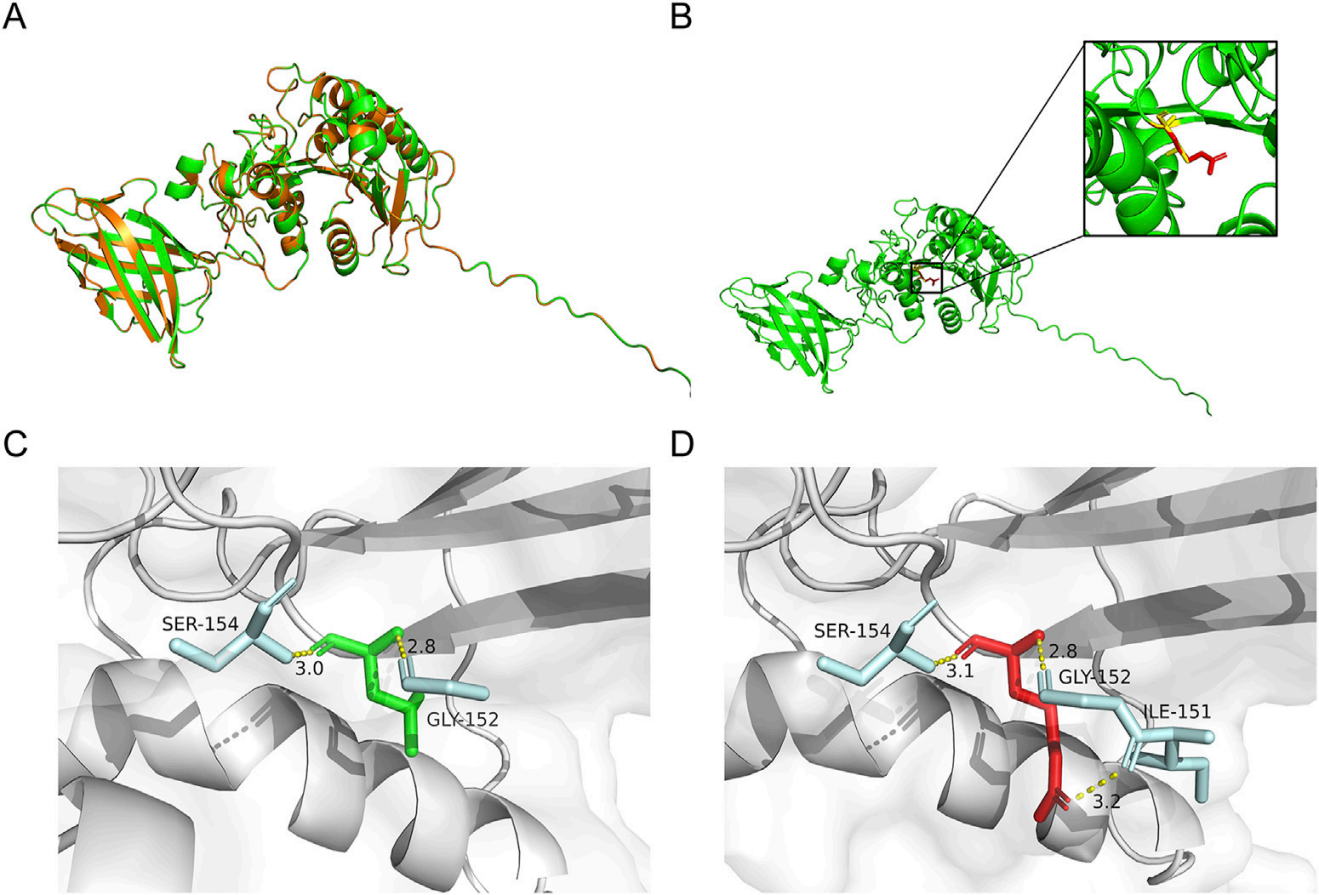
Predicted effects of variant on protein structure. **(A)** The superimposed view of the PA-PLA_1_α in its wild-type (WT) and p.Leu177Arg variant. **(B)** Predicted structures depicted the changes in PA-PLA_1_α after the Leu177Arg. Red and yellow structures separately indicate WT and the p.Leu177Arg variant. **(C,D)** The changes in hydrogen bonds between pre- and post-variant amino acids. Amino acids at position 177 of the WT and variant are shown in green and red, respectively. The amino acids involved in the interaction are highlighted in blue. The hydrogen bond is represented by the yellow dotted line.

The 3D structures of the WT and variant proteins were predicted to evaluate the influence of the novel p.Leu177Arg variant on the structure of the PA-PLA_1_α, based on an AlphaFold model (AF-Q8WWY8-F1-v4). The structures were visualized using PyMOL (Figure 2A). The substitution regions were highlighted in the protein model (Figure 2B). In the WT, Leu177 formed hydrogen bonds with Gly152 and Ser154 (Figure 2C). In the variant, the initial hydrogen bond distance between Leu177 and Ser154 increased from 3.0 Å to 3.1 Å, while an additional hydrogen bond formed between Leu177 and Ile151 (Figure 2D). The above-mentioned alterations likely reduce the stability and impact the intermolecular interactions of the protein.

### 3.5 Severe secretion defect of PA-PLA_1_α due to novel *LIPH* variant in ARWH

**FIGURE 3:**
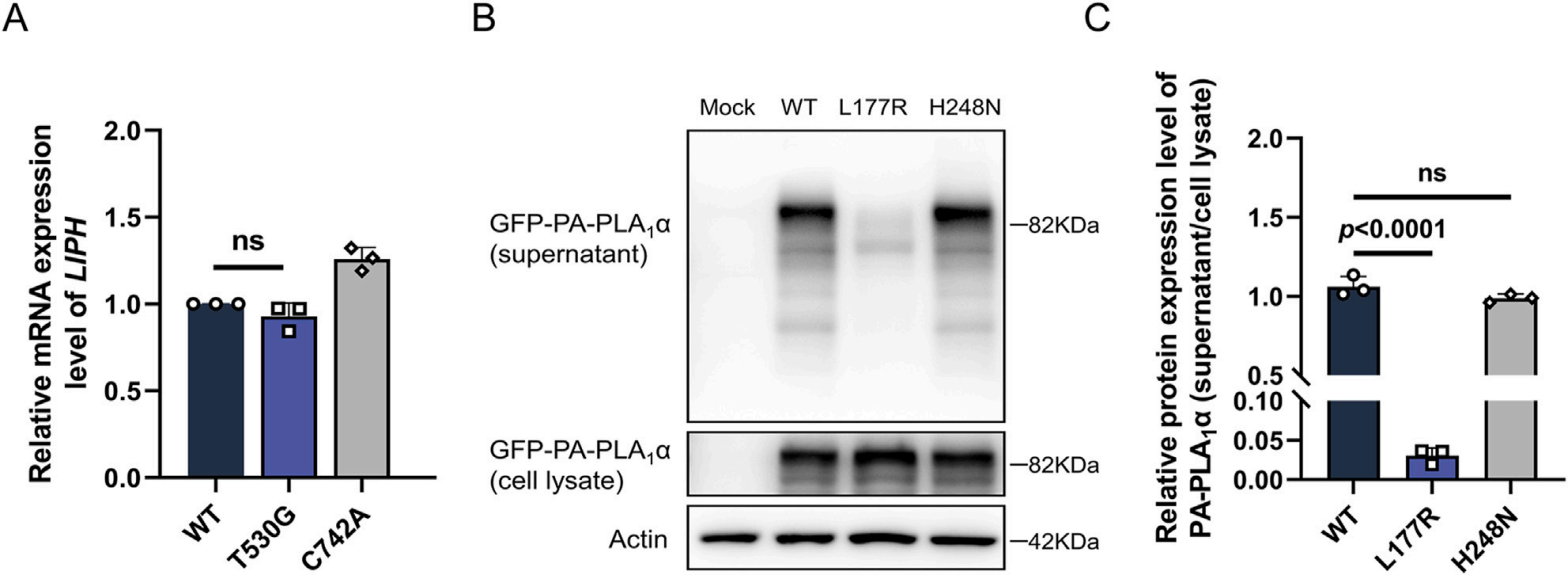
Functional analysis of the *LIPH* variants. **(A)** The relative mRNA expression level of *LIPH* in HEK293T cells transfected with the corresponding constructs was measured by qPCR. **(B,C)** Expression and secretion of WT, Leu177Arg, and His248Asn variant PA-PLA_1_α transfected in HEK293T cells were investigated using Western blotting. The top panel shows the expression levels of PA-PLA_1_α obtained from the constructs in the supernatant; the second panel demonstrates the expression levels of PA-PLA_1_α in the cell lysate. β-Actin expression was used for normalization.

PA-PLA_1_α functions as a secreted protein to perform its hydrolytic and catalytic activities. By transfecting the corresponding variants into HEK293T cells, we observed that the novel missense variant p.Leu177Arg did not affect mRNA expression or intracellular protein synthesis and expression (Figures 3A,B). However, the secretion assay revealed that the novel missense variant almost abolished the secretion of PA-PLA_1_α compared to the control (*p* < 0.0001), which would have resulted in a complete impairment of the hydrolysis function (Figures 3B,C). The p.His248Asn variant has been shown to cause a complete loss of PA-PLA_1_α hydrolytic activity, despite not affecting PA-PLA_1_α secretion (Shinkuma et al., 2010). In this study, we found that the novel variant directly led to a severe secretion defect of PA-PLA_1_α, resulting in impaired hydrolytic activity. Therefore, the novel compound heterozygous *LIPH* variants led to defective PA-PLA_1_α function, which is the underlying cause of the disease.

## 4 Discussion

A typical presentation of ARWH is marked by the occurrence of rough, dry, and curly spiral hair at birth (Akiyama, 2021; Hayashi and Shimomura, 2022). Hair growth is generally slow and sparse in affected individuals, with hairs that tend to break easily, resembling sheep’s wool, and varying degrees of sparsity, rarely exceeding a few inches in length (Shimomura, 2012). The majority of ARWH patients experience moderate-to-severe hypotrichosis, which varies among the individuals and families (Shimomura et al., 2009a; Takeichi et al., 2017). The worst situations result in the loss of all scalp hair (Shimomura et al., 2009b). Histopathological examination of the scalp reveals abnormal hair follicles and shafts. A few patients also exhibit thinning or lack of eyebrows, eyelashes, and beards, as well as armpit and body hair (Shimomura et al., 2009a).

According to Online Mendelian Inheritance in Man (OMIM), ARWH caused by *LIPH* variants is classified as ARWH2. To date, 34 pathogenic variants of *LIPH* associated with ARWH2 have been reported. The majority of ARWH2 families reside in Japan, Pakistan, and the Volga-Ural area of Russia, showing clear ethnic and geographic differences and a considerably high prevalence of their respective founder variants (Kazantseva et al., 2006; Shimomura et al., 2009a; Tanahashi et al., 2014; Hayashi and Shimomura, 2022). In the Japanese population specifically, the founder variants p.Cys246Ser or p.His248Asn have been reported in all ARWH2 families (Akiyama, 2021). Interestingly, according to data provided by HGMD, at least one of these two founder variants was consistently identified in all Chinese ARWH2 patients in this and previous studies (Table 1), providing factual evidence to support the hypothesis of genetic similarity between ARWH in Chinese and Japanese populations.

**TABLE 1:** *LIPH* variants found in Chinese patients with autosomal recessive woolly hair/hypotrichosis.

Patients	Years	*LIPH* variants	Amino acid changes	LIPH domain affected	Phenotypes	References
1	2014	c.742C>A	p.His248Asn	Lipase domain	Hypotrichosis	Liu LH et al.
2	2017	c.614A>G	p.His205Arg	Lipase domain	Woolly hair, hypotrichosis	Chang XD et al.
		c.742C>A	p.His248Asn			
3	2017	c.736T>A	p.Cys246Ser	Lipase domain	Woolly hair, hypotrichosis	
		c.742C>A	p.His248Asn			
4	2017	c.736T>A	p.Cys246Ser	Lipase domain	Woolly hair, hypotrichosis	
		c.742C>A	p.His248Asn			
5	2017	c.454T>A	p.Gly152Arg	Lipase domain	Woolly hair, hypotrichosis	
		c.742C>A	p.His248Asn			
6	2020	c.686delAins18	p.Asp229fs37*	Lipase domain	Woolly hair, hypotrichosis	Lv H et al.
		c.736T>A	p.Cys246Ser			
7	2022	c.736T>A	p.Cys246Ser	Lipase domain	Woolly hair, hypotrichosis	Qu B et al.
		c.742C>A	p.His248Asn			
8	2025	c.530T>G	p.Leu177Arg	Lipase domain	Woolly hair, hypotrichosis	This study
		c.742C>A	p.His248Asn			

Note: A comprehensive review of reported pathogenic *LIPH*, variants in Chinese patients with autosomal recessive woolly hair/hypotrichosis, including their corresponding protein domain and associated clinical phenotypes. Patient 3 and Patient 4 are two distinct individuals from the same family who share the same compound heterozygous *LIPH* variants.

PA-PLA_1_α, predominantly expressed in hair follicles, the anterior cortex, the cuticle of the hair shaft, and the Huxley layer of the inner root sheath (IRS), exerts its hydrolytic and catalytic activities as a secreted protein. Recent genetic studies on human hair disorders have demonstrated the essential role of LIPH-LPA-LPAR6 signaling in hair shaft development (Minakawa et al., 2024). It is proposed that the 2-acyl-LPA that PA-PLA_1_α generates in the IRS activates LPAR6 through either paracrine or autocrine mechanisms (Inoue et al., 2011). This activation induces the release of membrane-bound pro-TGFα through TACE-dependent mechanisms. Soluble TGFα then binds to epidermal growth factor receptor (EGFR) on IRS cells, activating EGFR and promoting IRS development for proper hair shaft formation. The p.His248Asn variant has been reported to cause a complete loss of PA-PLA_1_α hydrolytic activity and failure to activate LPAR6, despite not affecting PA-PLA_1_α secretion (Shinkuma et al., 2010). In this study, we found that the novel p.Leu177Arg variant directly led to a severe secretion defect of PA-PLA_1_α, resulting in impaired hydrolytic activity. Notably, such a severe secretion defect has only been observed in the p.Gly152Arg and p.His205Arg variants, with this the third instance (Chang et al., 2017). These findings suggest that variants resulting in the substitution of normal amino acids with arginine (Arg) may specifically impair PA-PLA_1_α secretion, as observed in p.Leu177Arg, p.Gly152Arg, and p.His205Arg. In contrast, other missense variants located near these three arginine-substitution sites, such as p.Cys246Ser, p.His248Asn, and even p.Ser154Ala—which is only one residue away from p.Gly152Arg—do not impact PA-PLA_1_α secretion (Shinkuma et al., 2010; Chang et al., 2017).

Arginine is particularly noteworthy due to its unique combination of physico-chemical properties, which distinguish it from most other amino acids. First, the side chain of arginine contains a guanidinium group, which endows it with a strong positive charge, making it the most positively charged residue among the 20 amino acids. This highly charged nature is unparalleled by other amino acids and allows arginine to participate in extensive ionic interactions and hydrogen bonding. However, in the context of protein folding, such strong electrostatic interactions can disrupt normal protein folding by affecting the stability of local structural elements (Harms et al., 2011; Perdriau et al., 2025). Second, arginine has one of the largest side chains among the 20 amino acids, and its bulkiness often introduces significant steric hindrance. This can lead to structural distortions, particularly when arginine is substituted in tightly packed regions (Gupta and Uversky, 2024). When investigating the impact of the p.Leu177Arg, p.His205Arg, and p.Gly152Arg variants, it becomes evident that these unique properties of arginine play a significant role. After arginine substitutions (p.Gly152Arg and p.His205Arg variants), the substituted arginine interacted with more nearby residues by hydrogen bonds (Supplementary Figures S1A–D), whereas other missense variants such as p.Ser154Ala and p.His248Asn tended to reduce interactions with other residues (Supplementary Figures S1E–H). Furthermore, arginine substitutions significantly increased residue size due to its bulky side chain. An increased number of interacting residues may work together with the bigger residue itself to form a larger group that disrupts the native conformation of the protein, causing steric hindrance and a loss of flexibility required for proper folding, ultimately resulting in endoplasmic reticulum (ER) retention and impaired secretion. The consistent observation across multiple variants suggests a potential mechanistic link between arginine substitutions and secretion defects. This observation provides important insights into the molecular mechanisms underlying protein secretion defects. Further research is required to confirm these findings.

Additionally, in this study, we conducted a literature review and comprehensively compiled the reported pathogenic variants of ARWH along with their corresponding phenotypes and populations (Supplementary Table S1). Our findings indicated that *LIPH* variants are the predominant cause of ARWH in Chinese patients, with no significant differences in phenotypic severity observed across different variants. In contrast, only one case has been associated with a *KRT25* variant, and no reported cases have been linked to *LPAR6* variants. Therefore, when clinically diagnosing Chinese patients who present with woolly hair and hypotrichosis, particular attention should be given to genetic screening for potential *LIPH* variants.

Despite the promising results of this study, several limitations should be acknowledged. First, while genetic and functional evidence strongly supports the pathogenicity of the *LIPH* variant, several predicted loss-of-function variants have been observed in public databases, such as GnomAD. However, in contrast to the other variants, which primarily affect the hydrolytic activity, the novel variant p.Leu177Arg identified here directly disrupts protein expression, leading to a complete loss of secretion, highlighting a less common pathogenic mechanism. Second, our finding is based on a single patient. Future investigations involving larger cohorts would be essential to validate these findings and better clarify the genotype-phenotype correlations of *LIPH* variants.

In conclusion, this study reported a patient with ARWH and confirmed the clinical diagnosis through *in vitro* experiments, demonstrating that the novel *LIPH* c.530T>G (p.Leu177Arg) variant affected protein function by impairing PA-PLA_1_α secretion. This direct effect on protein secretion is extremely rare in previous studies of ARWH and may be related to arginine substitutions, providing new insights into the pathogenic mechanism of ARWH. Our findings expand the spectrum of *LIPH* variants associated with ARWH of Chinese origin and have the potential to influence the formulation of new diagnostic and treatment approaches for this condition.

## Data Availability

The data presented in the study are deposited in the Figshare repository, https://doi.org/10.6084/m9.figshare.28853567.
