# Supplementary material for: Complete defect in PA-PLA1α secretion function leading to autosomal recessive woolly hair and hypotrichosis: insights from a novel compound heterozygous LIPH variant study in a Chinese pedigree
Source: Front Genet. 2025 May 9;16:1591409. doi: 10.3389/fgene.2025.1591409 (PMC12098026; doi:10.3389/fgene.2025.1591409)
Supplement: Supplementary file 1 [file DataSheet1.pdf]

Supplementary Material

1.1 Supplementary Figure

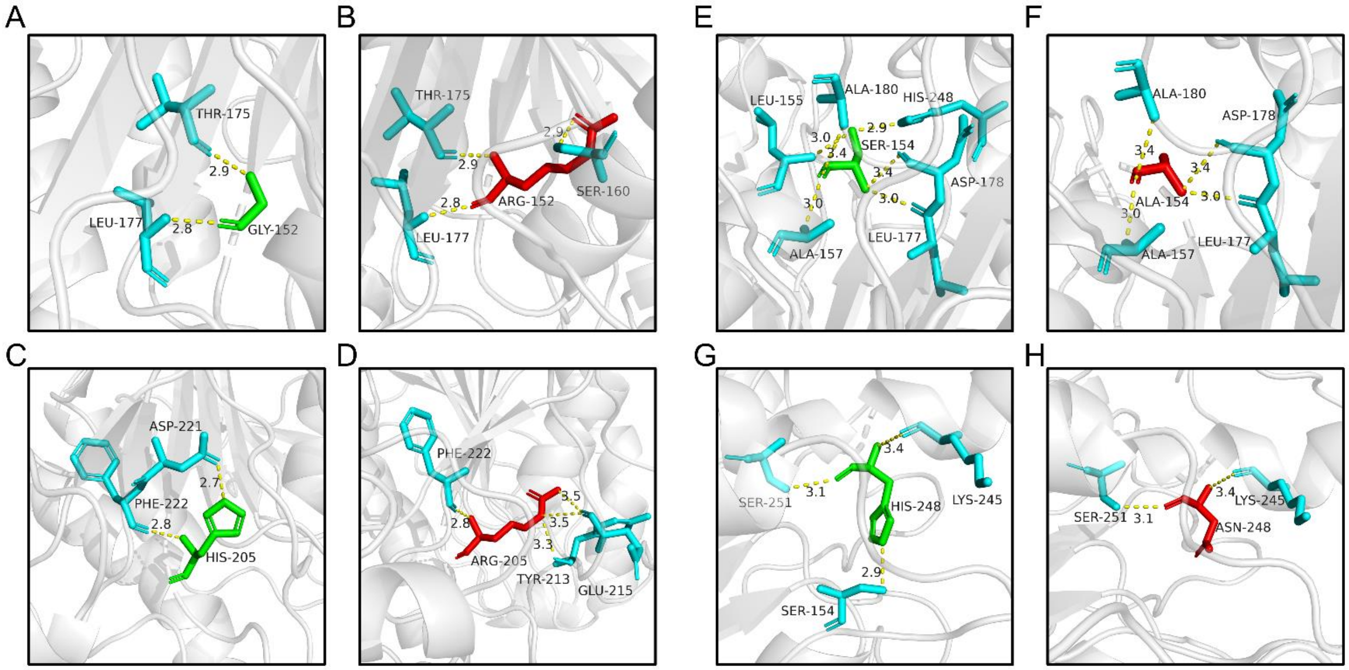

Supplementary Figure 1. Predicted effects of arginine substitution variants and other missense variants on protein structure. (A-D) Structural changes in PA-PLA$_1$α before and after the two arginine substitution variants: p.G152R (A-B) and p.H205R (C-D). Notably, both variants resulted in an increased number of interacting residues, linked by hydrogen bonds. (E-H) Structural changes in PA-PLA$_1$α before and after the two missense variants: p.S154A (E-F) and p.H248N (G-H). Notably, both variants resulted in fewer interacting residues. Amino acids of the wild-type and variant are shown in green and red, respectively. Amino acids involved in interactions are highlighted in blue, and hydrogen bonds are represented by yellow dotted lines.

1.2 Supplementary Table

Table S1. Summary of reported pathogenic variants associated with autosomal recessive woolly hair/hypotrichosis

Phenotypes (OMIM No.)	Genes	Variant types	Variants	Amino acid changes	Clinical phenotypes	Populations
ARWH1 (#278150)	*LPAR6*	Missense/nonsense	c.8G>C	p.Ser3Thr	Hypotrichosis	Pakistani, Japanese
			c.47A>T	p.Lys16Met	Woolly hair, hypotrichosis	Pakistani
			c.188A>T	p.Asp63Val	Woolly hair	Pakistani
			c.436G>A	p.Gly146Arg	Hypotrichosis	Pakistani, Iranian
			c.463C>T	p.Gln155Term	Hypotrichosis	Saudi Arabian
			c.562A>T	p.Ile188Phe	Woolly hair, hypotrichosis	Pakistani
			c.565G>A	p.Glu189Lys	Woolly hair	Pakistani, United Arab Emirates, Japanese
			c.587C>T	p.Pro196Leu	Hypotrichosis	Pakistani
			c.734A>G	p.Tyr245Cys	Woolly hair	Pakistani
			c.736A>G	p.Asn246Asp	Woolly hair	Syrian, Spanish, Turkish

			c.742A>T	p.Asn248Tyr	Hypotrichosis	Pakistani, Japanese
			c.756T>A	p.Tyr252Term	Hypotrichosis	Japanese
			c.830T>C	p.Leu277Pro	Hypotrichosis	Pakistani, Japanese
			c.833G>A	p.Cys278Tyr	Woolly hair	Brazilian, Japanese
			c.859G>C	p.Asp287His	Woolly hair, hypotrichosis	Spanish
		Small deletions	c.329_331delTTA	p.(Asp13Argfs*4)	Hypotrichosis	Pakistani
			c.373_374delAA	p.(Phe24Hisfs*29)	Woolly hair	Pakistani
			c.472delC	p.(Thr54Asnfs*5)	Hypotrichosis	Pakistani
			c.669_672delCAAA	p.(Ile195Tyrfs*11)	Hypotrichosis	Pakistani
		Small indels	c.172_177delAACTTGinsT	p.(Asn58Cysfs*31)	Woolly hair	Pakistani
			c.409_426del18insC	p.(Cys137Hisfs*20)	Woolly hair	Pakistani
		Gross deletions	c.-689+858_*8757del12211	/	Woolly hair, hypotrichosis	Turkish

		Gross insertions	4156 bp, 248 bp upstream from transcription start site	/	Hypotrichosis	Japanese
ARWH2 (#604379)	*LIPH*	Missense/nonsense	c.2T>C	p.Met1Thr	Hypotrichosis	Pakistani
			c.179C>G	p.Ser60Term	Woolly hair, hypotrichosis	Lebanese
			c.322T>C	p.Trp108Arg	Hypotrichosis	Pakistani
			c.328C>T	p.Arg110Term	Hypotrichosis	Pakistani
			c.454G>A	p.Gly152Arg	Woolly hair, hypotrichosis	Chinese
			c.530T>G	p.Leu177Arg	Woolly hair, hypotrichosis	Chinese, this study
			c.614A>G	p.His205Arg	Woolly hair, hypotrichosis	Chinese
			c.619G>C	p.Asp207His	Woolly hair, hypotrichosis	Japanese
			c.671C > G	p.Pro224Arg	Woolly hair, hypotrichosis	Japanese
			c.688C>T	p.Gln230Term	Hypotrichosis	Pakistani
			c.699C>G	p.Cys233Trp	Woolly hair, hypotrichosis	Japanese
			c.736T>A	p.Cys246Ser	Woolly hair, hypotrichosis	Japanese, Chinese

			c.742C>A	p.His248Asn	Woolly hair, hypotrichosis	Japanese, Chinese
			c.778A>T	p.Arg260Term	Hypotrichosis	Pakistani
			c.886G>T	p.Gly296Cys	Hypotrichosis	Qatari
		Splicing	c.417+1G>C	48 aa deletion	Woolly hair	Japanese
			c.629-1G>C	Skipping of exon 5	Hypotrichosis	Pakistani
			c.982+5G>T	p.Met328Serfs41*	Woolly hair	Japanese
			c.1095-3C>G	p.Glu366IIefs7*	Woolly hair, hypotrichosis	Japanese
		Small deletions	c.346_350delATATA	p.(Ile116Tyrfs*5)	Hair growth deficiency	Pakistani
			c.624delT	p.(Asp209Metfs*9)	Hypotrichosis	Pakistani
			c.659_660delTA	p.(Ile220Argfs*25)	Hypotrichosis	Pakistani, Guyanese
			c.683delT	p.(Leu228Trpfs*32)	Hypotrichosis	Pakistani
			c.932delC	p.(Pro311Leufs*3)	Hypotrichosis	Pakistani
		Small insertions	c.404_410dupTTGACCA	p.(Gln137Hisfs*2)	Hypotrichosis	Austrian

			c.558_559insT	p.(Lys187*)	Woolly hair, hypotrichosis	Japanese
			c.1303_1309dupGAAAACG	p.(Val437Glyfs*4)	Hypotrichosis	Guyanese
		Small indels	c.460_461delAGinsGA	p.(Ser154Asp)	Woolly hair	Japanese
			c.620_627delACACTGATins14	p.(Asp207_Asp209delinsAlaProPheLeuVal)	Hypotrichosis	Italian
			c.686delAins18	p.(Asp229Glyfs*37)	Woolly hair, hypotrichosis	Japanese, Chinese
			c.982+2_982+19del18insAGA	/	Woolly hair, hypotrichosis	Japanese
		Gross deletions	Ex4(c.527_628)del	176G-209D (34 aa) deletion	Hair growth deficiency	Mari and Chuvash populations in Russia
			c.886+405_1094+962del5290	p.(Gly296Valfs*12)	Hypotrichosis	Pakistani
		Gross insertions	c.280_369dup	p.Gly94_Lys123dup	Hypotrichosis	Central European, Israeli families of Arab Muslim descent, Turkish family of Aramaic-Christian,

						Austrian, Jewish, Arab, Pakistani
ARWH3 (#616760)	*KRT25*	Missense/nonsense	c.266G>A	p.Arg89His	Woolly hair, hypotrichosis	Pakistani
			c.712G>T	p.Val238Leu	Woolly hair, hypotrichosis	Volga–Ural region of Russia
			c.950T>C	p.Leu317Pro	Woolly hair	Pakistani
			c.1127T>G	p.Leu376Arg	Woolly hair, hypotrichosis	Chinese
– (*611956)	*C3orf52*	Missense/nonsense	c.34G>T	p.Glu12Term	Hypotrichosis	Arab Muslim descent
			c.492T>G	p.Tyr164Term	Hypotrichosis	Hispanic descent

Note: A comprehensive review of reported pathogenic variants in patients with autosomal recessive woolly hair/hypotrichosis, including their clinical phenotypes and population characteristics. Abbreviations: ARWH1, woolly hair, autosomal recessive 1, ARWH2, A woolly hair, autosomal recessive 2, ARWH3, woolly hair, autosomal recessive 3.
